# Evaluating AI diagnostic accuracy in approximal dental caries detection on bitewing radiographs

**DOI:** 10.1007/s00784-026-06882-z

**Published:** 2026-04-29

**Authors:** Kostis Giannakopoulos, Argyro Kavadella, Dimitrios Paraskevis, Aristidis Arhakis, Miltiadis A. Makrygiannakis, Eleftherios G. Kaklamanos

**Affiliations:** 1 School of Dentistry, European University Cyprus, 6 Diogenous str, Nicosia, 2404 Cyprus; 2 Medical School, National and Kapodistrian University of Athens, 75 Mikras Asias str, Athens, 11527 Greece; 3 School of Dentistry, Aristotle University of Thessaloniki, Aristotle University of Thessaloniki Campus, Thessaloniki, 54124 Greece; 4 School of Dentistry, National and Kapodistrian University of Athens, 2 Thivon str, Athens, 11527 Greece; 5 Hamdan Bin Mohammed College of Dental Medicine, Mohammed Bin Rashid University of Medicine and Health Sciences, P.O. Box: 505055, Dubai Healthcare City, Dubai United Arab Emirates

**Keywords:** Artificial intelligence, Bitewing radiographs, Caries detection, Diagnostic accuracy, Computer-aided diagnosis

## Abstract

**Objectives:**

To evaluate the diagnostic accuracy of the Diagnocat™ artificial intelligence (AI) system for caries detection on bitewing radiographs compared with expert human examiners, with emphasis on differences between enamel and dentin lesions.

**Materials and methods:**

A sample of 100 digital bitewing radiographs (1540 surfaces) was retrospectively selected from the European University Cyprus dental clinic database using a systematic backward screening method. Radiographs were obtained with a standardized phosphor plate system and anonymized before analysis. Two independent experts (operative dentistry and oral radiology) established the reference standard. AI and human assessments were binarized (caries/no caries; enamel/dentin) and compared. Sensitivity, specificity, positive predictive value (PPV), negative predictive value (NPV), and accuracy were calculated, and statistical significance was tested across detection categories.

**Results:**

Diagnocat™ showed high specificity (94.3%, 95% CI: 92.4%–96.0%) and NPV (96.1%, 95% CI: 94.7%–97.3%), with an overall accuracy of 91.6% (95% CI: 89.7%–93.4%). Sensitivity was moderate (73.1%, 95% CI: 65.9%–79.9%), and PPV was 64.7% (95% CI: 57.7%–71.5%). Agreement with the expert consensus was substantial (Cohen’s κ = 0.638). For enamel lesions, sensitivity and specificity were 73.3% (95% CI: 62.8%–82.7%) and 92.9% (95% CI: 91.0%–94.7%) with moderate agreement with the consensus (Cohen’s κ = 0.492) and for dentin lesions they were 72.8% (95% CI: 61.8%–83.8%) and 92.8% (95% CI 90.9%–94.6%) with moderate agreement with the consensus (Cohen’s κ = 0.468). NPV remained high (≥ 98.0%), while PPV was low (42.0% and 39.2%), across lesion types. Detection patterns differed significantly between AI and the reference standard (*p* < 0.001).

**Conclusions:**

Diagnocat™ demonstrated good diagnostic performance in ruling out caries. However, its overall lower sensitivity emphasizes the need for clinician oversight, especially in detecting early-stage disease.

**Clinical relevance:**

This study offers an independent validation of Diagnocat™ using bitewing radiographs. It demonstrates lesion-depth–specific insights, showing that while AI is highly reliable for excluding disease, its predictive value remains limited.

**Supplementary Information:**

The online version contains supplementary material available at 10.1007/s00784-026-06882-z.

## Introduction

 Artificial intelligence (AI) is increasingly transforming dentistry by enhancing diagnostic accuracy, supporting treatment planning, and improving radiographic interpretation [1, 2]. Modern AI systems, particularly deep learning models, can analyze dental data (e.g. radiographs, photographs, chart notes) rapidly and consistently, assisting clinicians in decision-making. Several studies in medical applications, where AI algorithms have begun to match or even surpass human experts in certain diagnostic tasks [3–5].

Dental caries remains a prevalent chronic disease worldwide, necessitating early detection for effective management and prevention. Traditional methods rely primarily on visual examination and radiographic interpretation, with bitewing radiographs considered the gold standard for proximal caries detection. The interpretation of radiographs has traditionally depended on the clinician’s expertise, training, and experience. However, the interpretation of these radiographs is subject to inter- and intra-observer variability, potentially leading to inconsistent diagnoses [6, 7].

Standardized assessment systems, such as the International Caries Detection and Assessment System (ICDAS), have improved the consistency of caries diagnosis [8]. Ekstrand et al. demonstrated strong correlation between ICDAS radiographic criteria and histological findings, validating ICDAS as a standardized assessment tool [9].

In dental radiology, AI applications now span bitewing, periapical, panoramic and cone-beam computed tomography (CBCT) images for detecting different pathologies. Deep learning algorithms, particularly CNNs, have shown remarkable potential and several studies show promising results. Casalegno et al. developed a deep learning model that holds promise for increasing the speed and accuracy of caries detection [10]. Similarly, Schwendicke et al. conducted a systematic review showing that AI systems achieved accuracy of 0.82–0.89 in caries detection [11] and several recent studies show better detection accuracy by neural networks than by dentists [12]. Lee et al. evaluated a CNN that achieved up to 89% accuracy, 92% sensitivity and 94% specificity in detecting dental caries in premolars and molars [13] while Devito et al. reported significantly better performance of a CNN than human observers [14]. Two recent meta-analyses reported pooled sensitivity and specificity of approximately 0.94/0.91 and 0.87/0.89 respectively, for AI systems detecting proximal caries on bitewing radiographs [15, 16]. These models often exceed human dentists in sensitivity for early lesions, while specificity is comparable or slightly lower [17].

Diagnocat™ (Diagnocat Co. Ltd., San Francisco CA, USA) is a commercially available, cloud-based AI software that uses CNNs to automatically detect and label dental pathologies, including primary and secondary caries, on radiographic images. Previous studies evaluating Diagnocat™ have reported moderate to substantial agreement with expert examiners and diagnostic performance broadly comparable to that of trained clinicians. Other notable systems include Denti.AI, and deep learning frameworks such as ResNet, AssistDent and YOLO, which have shown high precision for dental findings in research settings. Overall, comparative studies suggest that AI can significantly assist dentists in caries diagnosis but should not yet be used independently [18–24]. However, these studies have largely been conducted using internally curated or non-independent datasets.

From both methodological and clinical perspectives, external validation using independent image samples is essential to assess the robustness, reproducibility, and real-world applicability of AI-based diagnostic systems prior to widespread clinical implementation. Performance may vary when algorithms are applied to datasets acquired under different clinical conditions or evaluated against independent expert reference standards.

Therefore, the present study aims to externally validate the diagnostic performance of the Diagnocat™ AI software for detecting dental caries on bitewing radiographs, using an independent image dataset, in comparison with human observers. By assessing sensitivity and specificity relative to expert consensus reference standard, this study will help determine if Diagnocat™ can reliably replicate expert radiographic caries diagnosis. The null hypothesis is that there is no difference in caries detection performance between experienced clinicians and the Diagnocat™ AI system.

## Materials and methods

This diagnostic accuracy study adhered to ethical guidelines for diagnostic research and the European University Cyprus Institutional Committee on Ethics and Bioethics approved the protocol (EUC ETHICS COMMITTEE 2025-1). The study was designed and reported in accordance with the Standards for Reporting Diagnostic Accuracy Studies (STARD 2015) guidelines.

### Sample size calculation, selection criteria, and technical aspects

 A sample of 100 digital bitewing radiographs was selected from the imaging database of the European University Cyprus dental clinic, comprising 1540 approximal surfaces and included posterior teeth with various types of dental restorations and differing levels of caries lesions, ensuring a clinically diverse dataset.

Radiographs were selected using a systematic backward screening method: a fixed date was defined, and all bitewing radiographs taken before this date were retrieved in reverse chronological order. Each image was assessed sequentially according to the predefined inclusion and exclusion criteria. Radiographs that did not meet the criteria were excluded, and screening continued backwards until 100 eligible radiographs had been identified. This approach ensured a transparent and reproducible selection process, minimized selection bias, and ensured that all radiographs taken before the index date had an equal opportunity for inclusion, contingent solely on meeting the eligibility criteria.

Inclusion criteria were: (i) radiographs displayed posterior teeth (premolars and/or molars) with clearly visible approximal and occlusal surfaces; (ii) demonstrated acceptable diagnostic quality, including appropriate exposure, contrast, and no motion artefacts; (iii) showed non-overlapped interproximal contacts; (iv) contained teeth with no restorations or with restorations that still allowed clear assessment of the surfaces for potential lesions; (v) represented standard bitewing projections with correct geometric characteristics.

Exclusion criteria were: (i) radiographs exhibited technical errors such as cone-cut, motion blur, or cropping that impaired diagnostic interpretation; (ii) presented overlapping interproximal contacts that obscured evaluation of interproximal caries; (iii) included teeth with extensive metallic restorations, or other structures that obstructed assessment of the surfaces of interest; (iv) reflected non-standard projections or incorrect angulation that distorted tooth anatomy and prevented reliable assessment; (v) were radiographs of pediatric patients depicting deciduous or mixed dentition.

Power analysis was not performed because of the absence of prior data on the expected differences between the two diagnostic methods. Nevertheless, the inclusion of 1,540 examined surfaces was considered sufficient to allow detection of potential differences in the diagnostic accuracy of the AI method at the surface level. All bitewing radiographs were acquired using the Gendex GX-770 x-ray machine, operating at 70kVp, 7 mA (Gendex Corp, Milwaukee, Wisconsin, USA), using VistaScan phosphor imaging plates size 2 and the VistaScan Nano Easy scanner (Dürr Dental SE, Bietigheim-Bissingen, Germany). Images were exported in JPEG format and anonymized before analysis.

### Reference standard

 A reference standard dataset for caries presence was established by consensus between two experienced university faculty members: an Associate Professor of Operative Dentistry (KG) and an Assistant Professor of Oral and Maxillofacial Radiology (AK), with over 25 years of clinical experience. All radiographs were viewed under ambient light, using a Dell Latitude 3420 Laptop (Dell Inc, Texas, United States), on a 14-inch display screen with a 1360 × 768 resolution, allowing the display of 256 shades of grey (8-bit system). Viewers were allowed to perform image manipulation processes, such as magnification and/or contrast / brightness adjustment, as they would do in the clinical setting to enhance their diagnostic accuracy. First, each observer independently examined the bitewing radiographs to identify proximal caries lesions. Occlusal primary and secondary (adjacent to restorations) radiolucencies were not included in the analysis. The identified tooth surfaces, caries presence or absence, and ICDAS radiographic scores per surface [8], as well as the total number of lesions per radiograph, were recorded on a spreadsheet. For the statistical analysis, ICDAS scores RA1 and RA2 (lesions confined to enamel), were grouped as “enamel lesions” and scores RA3–RC6 (lesions extending into dentin), were grouped as “dentin lesions”, following conventions used in radiographic ICDAS-based validation studies [25, 26]. Before the study, both observers underwent a calibration session, reviewing sample radiographs and aligning on ICDAS diagnostic criteria to ensure consistency. The assessments were conducted blinded to each assessor’s judgment and patient clinical information, providing an unbiased diagnostic evaluation.

Following the independent evaluations, any disagreements were resolved during a joint consensus session. All radiographs with discrepant assessments were re-evaluated by the observers until agreement was reached, thereby ensuring that no indeterminate classifications remained. The resulting consensus diagnosis served as the reference standard. The agreement between the two expert clinicians (inter-observer agreement) was assessed. Inter-observer agreement statistics reported in this study refer to the independent pre-consensus evaluations and were included to document examiner calibration prior to consensus formation.

### Test method

Following the development of the reference standard, all radiographs were analyzed with Diagnocat™ using the same laptop computer as during the viewing process. This software was selected because it represents a commercially available, clinically oriented system, allowing assessment of AI performance under realistic conditions relevant to routine clinical workflows. It has been extensively investigated in similar studies for estimating the diagnostic accuracy of both 2D and 3D images [21, 27–30].

**Fig. 1:**
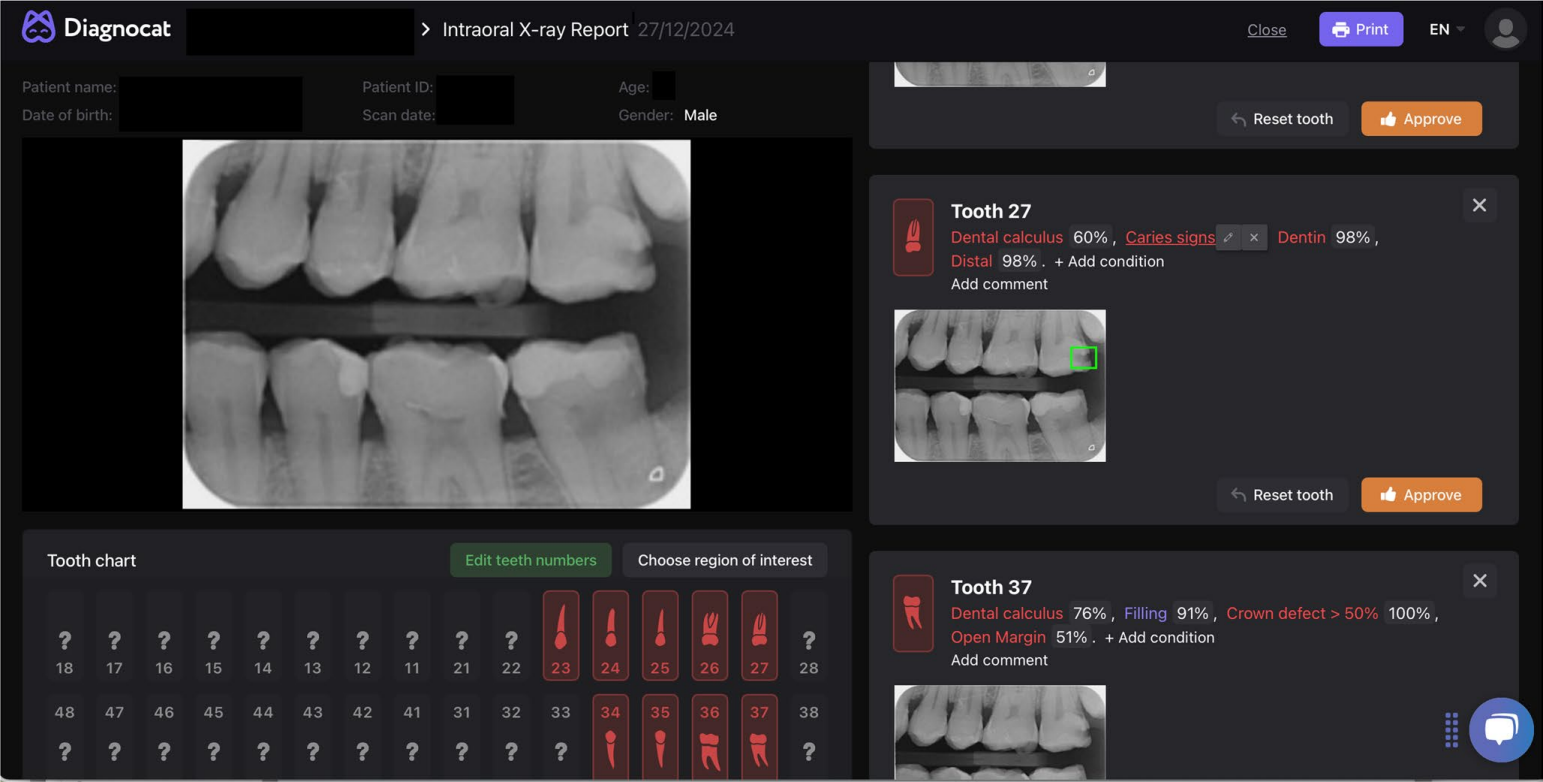
Diagnocat™ output for a bitewing radiograph showing automatic detection of caries lesions. This visualization reflects how the AI software highlights potential caries-related findings. Green-colored overlay highlights the suspected area of decay, with associated probability scores displayed. All other detected pathologies by the software were disregarded for the purposes of this study

**Fig. 2:**
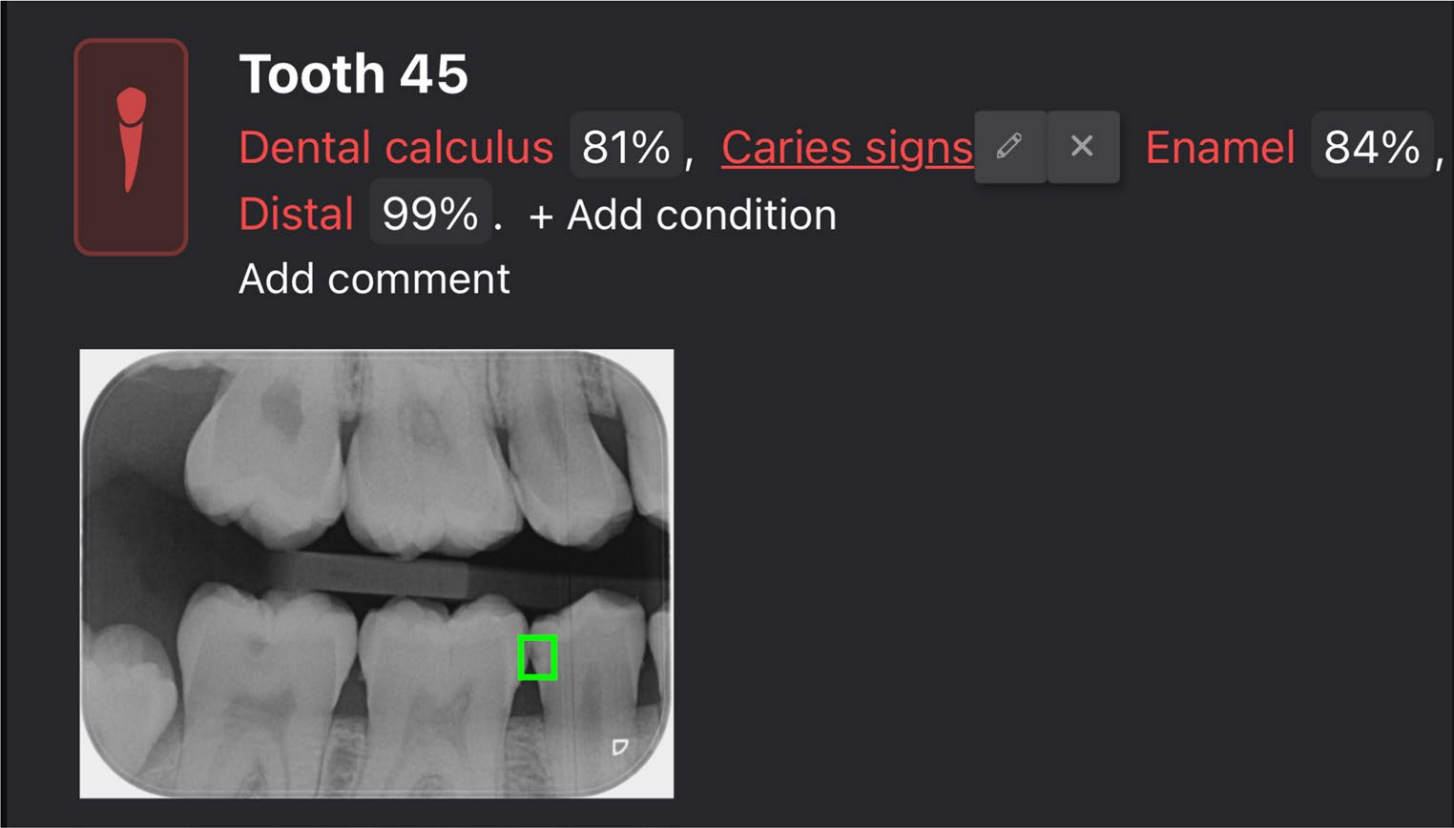
Example of Diagnocat™ classification of detected caries on a bitewing radiograph. Lesions are categorized by the software into “enamel”, “dentin”, or “secondary caries,” with visual color-coded overlays and accompanied by probability scores. This classification is clinically relevant, as differentiation between the depth of enamel and dentin lesions informs treatment planning and risk assessment

The version used was fully locked and trained before this evaluation, with no additional training on our sample. The software detects various dental pathologies on each tooth and assigns a probability score to each detected finding. For the purposes of the present study, only caries lesions with software-assigned probability scores ≥ 50% were considered positive. The study evaluated the software using its default diagnostic output, and probability scores were not exported separately for threshold optimization analyses. All other detected pathologies were disregarded (Fig. 1). For each radiograph, the software-generated output included colored overlays indicating suspected caries areas and classified lesions into “enamel” and “dentin”, caries (Fig. 2). The outcome of the AI assessment for each tooth surface was binarized as “caries” vs. “no caries” and as “enamel lesions” and “dentin lesions” to allow for direct comparison with the human reference standard. The lesions reported by the software were registered in a spreadsheet.

### Statistics

The agreement between the two expert clinicians was assessed using Cohen’s Kappa. All paired comparisons of proportions were conducted using a cluster-robust McNemar-type approach with patient as the clustering unit.

Diagnostic accuracy metrics were calculated separately for three binary outcome definitions: any caries, enamel lesions only, and lesions extending to dentin. We considered a match between two different assessments if tooth number, tooth surface and lesion depth were identical. From these values we calculated the sensitivity, specificity, positive predictive value (PPV), and negative predictive value (NPV), for the AI software in detecting caries. The 95% confidence intervals were estimated using a patient-level cluster bootstrap. The agreement between the reference standard dataset and AI software assessment was evaluated using Cohen’s Kappa. All paired comparisons of proportions were conducted using a cluster-robust McNemar-type approach with patient as the clustering unit. All statistical analyses were performed using IBM SPSS v.29.0. The significance level for all hypothesis testing procedures was set at α = 0.05 (*p* ≤ 0.05).

## Results

The two experts independently evaluated all radiographs prior to consensus formation. Positive and negative diagnoses regarding the identification of caries lesions on each surface are presented in Table 1. Cohen’s Kappa was 0.8096 for the detection of caries, indicating an almost perfect level of agreement between the assessors. Moreover, using the cluster-robust McNemar-type analysis, the mean paired difference was 0.0091 (SE: 0.006), which was not statistically significant (two-sided hypothesis; *p* = 0.16).

**Table 1:** Positive and negative diagnoses regarding the identification of caries lesions on each surface by the two expert clinicians

Any caries lesion	Assessor 2		
Assessor 1	Positive	Negative	**Subtotal**
Positive	154	38	192
Negative	24	1324	1348
Subtotal	178	1362	**1540**

Cohen’s kappa = 0.80959

Compared to the reference standard dataset, the positive and negative diagnoses regarding identifying caries lesions on each surface by the AI software are presented in Table 2. Cohen’s Kappa coefficient was 0.638 for identifying any caries, indicating substantial agreement. Accounting for clustering of surfaces within patients using the cluster-robust McNemar-type analysis, the mean paired difference was 0.016 (SE: 0.009). This difference was not statistically significant under a two-sided hypothesis (*p* = 0.088).

**Table 2:** Positive and negative diagnoses regarding the identification of caries lesions on each surface by the AI software compared to the reference standard dataset

Any caries lesion	AI software assessment		
Reference standard dataset	Positive	Negative	**Subtotal**
Positive	141	52	193
Negative	77	1270	1347
Subtotal	218	1322	**1540**

The sensitivity and specificity were 73.1% (95% CI 65.9%–79.9%) and 94.3% (95% CI 92.4%–96.0%), respectively, while the PPV and NPV were 64.7% (95% CI: 57.7%–71.5%) and 96.1% (95% CI: 94.7%–97.3%), respectively (Table 3). Overall agreement (accuracy) was 91.6% (95% CI: 89.7%–93.4%). Accordingly, the null hypothesis of no difference in lesion counts between the AI and the reference standard was rejected.

**Table 3:** Diagnostic performance of the AI software for (i) any caries (enamel + dentin combined) and (ii) depth-specific caries detection (enamel and dentin analyzed separately), compared with the reference standard dataset. Sensitivity, specificity, positive predictive value (PPV), and negative predictive value (NPV) with 95% confidence intervals are shown

Comparisons	Sensitivity (95% CI)	Specificity (95% CI)	Positive predictive value (95% CI)	Negative predictive value (95% CI)
Identification of caries lesions by AI software compared to the reference standard dataset (Overall caries lesions)	73.1% (95% CI: 65.9%–79.9%)	94.3% (95% CI 92.4%–96.0%)	64.7% (95% CI 57.7%–71.5%)	96.1% (95% CI 94.7%–97.3%)
Identification of enamel caries lesions by AI software compared to the reference standard dataset (Subset analysis)	73.3% (95% CI 62.8%–82.7%)	92.9% (95% CI 91.0%–94.7%)	42.0% (95% CI 33.1%–51.2%)	98.0% (95% CI 97.1%–98.8%),
Identification of dentin caries lesions by AI software compared to the reference standard dataset (Subset analysis)	72.8% (95% CI 61.8%–83.8%)	92.8% (95% CI 90.9%–94.6%)	39.2% (95% CI 31.6%–46.6%)	98.2% (95% CI 97.2%–99.0%)

The positive and negative diagnoses regarding identifying enamel caries lesions on each surface by the AI software, compared to the reference standard dataset, are presented in Table 4. Cohen’s Kappa coefficient was 0.492 for identifying enamel caries lesions, indicating a moderate level of agreement. Using the cluster-robust McNemar-type analysis, the mean paired difference was 0.0487 (SE: 0.0096). This difference was statistically significant (two-sided hypothesis; *p* < 0.001). However, the proportions of carious and non-carious surfaces differed significantly (*p* < 0.001). The sensitivity and specificity were 73.3% (95% CI 62.8%–82.7%) and 92.9% (95% CI 91.0%–94.7%), respectively, while the PPV and NPV were 42.0% (95% CI 33.1%–51.2%) and 98.0% (95% CI 97.1%–98.8%), respectively (Table 3). Overall agreement was 91.6% (95% CI 89.7%–93.4%).

**Table 4:** Confusion matrices showing agreement between AI system assessment and the reference standard for enamel and dentin caries lesions

Enamel caries lesions			
Reference standard dataset	AI Positive	AI Negative	**Subtotal**
Positive	74	27	101
Negative	102	1337	1439
Subtotal	176	1364	**1540**

Dentin caries lesions			
Reference standard dataset	AI Positive	AI Negative	**Subtotal**
Positive	67	25	92
Negative	104	1344	1448
Subtotal	171	1369	**1540**

The positive and negative diagnoses regarding identifying dentin caries lesions on each surface by the AI software, compared to the reference standard dataset, are presented in Table 4. Cohen’s Kappa coefficient was 0.468 for identifying dentin caries lesions, indicating a moderate level of agreement. Using the cluster-robust McNemar-type analysis, the mean paired difference was 0.0513 (SE: 0.0098). This difference was statistically significant (two-sided hypothesis; *p* < 0.001). The sensitivity and specificity were 72.8% (95% CI 61.8%–83.8%) and 92.8% (95% CI 90.9%–94.6%), respectively, while the PPV and NPV were 39.2% (95% CI 31.6%–46.6%) and 98.2%, respectively (Table 3). Overall agreement was 91.6% (95% CI 89.7%–93.4%).

## Discussion

This diagnostic accuracy study compared Diagnocat™ with expert consensus on 1,540 approximal tooth surfaces from 100 bitewing radiographs, using surface-level labels (enamel vs. dentin) to align with the AI’s output. Overall, the AI software achieved substantial agreement with the reference standard for “any caries” (Cohen’s κ = 0.638) and high specificity (~ 94.3%) alongside moderate sensitivity (~ 73.1%), producing a high NPV (~ 96.1%) but a lower PPV (~ 64.7%). Specificity was high (~ 94.3%), meaning the AI correctly recognized healthy surfaces most of the time, with relatively few false positives. The high NPV, implies that when the AI labels a surface as caries-free, it is very likely to be truly healthy; however, this must be interpreted alongside the low PPV, which indicates that a substantial proportion of AI-positive findings may represent false positives. Importantly, the proportions of positive/negative classifications differed significantly between AI and the reference standard (cluster-robust McNemar-type analysis *p* = 0.04), and the per-radiograph lesion counts also differed, indicating a systematic tendency of the AI to over-flag some features as carious compared with expert adjudication. By contrast, the two expert examiners showed very high inter-examiner agreement (Cohen’s κ = 0.809), with no significant differences in positive/negative proportions or per-image lesion counts, supporting the robustness of the consensus reference standard [7]. Although formal intra-observer repeatability was not reassessed, prior calibration and structured consensus procedures were implemented to minimize intra-rater variability [31].

The overestimation of disease presence clinically, poses a serious risk of overtreatment, as this may result in unnecessary interventions for lesions that do not require treatment [32]. It was reported that AI usage for caries detection increased the treatment intensity, as significantly more enamel caries lesions were detected and managed non-/micro-invasively or invasively [32]. Consequently, false-positive diagnoses can compromise the cost-effectiveness of caries detection and treatment, as unnecessary treatment of initially healthy teeth can increase the likelihood of more extensive interventions over time [33]. Dentists must critically interpret the findings of the algorithm while using their inherent higher specificity, and combine them with the clinical examination before initiating any irreversible intervention [12, 33]. It is important to note that this AI software provides a continuous probability estimate for the presence of caries. For the purposes of binary classification, a 50% probability threshold was selected, corresponding to the default operational setting of the Diagnocat™ platform, in the absence of an externally validated, clinically endorsed alternative cutoff. While higher thresholds (e.g., 80–90%) would be expected to improve specificity, such thresholds would need to be clinically validated and calibrated against patient-level outcomes. The present study therefore focuses on diagnostic performance benchmarking rather than optimization of clinical decision thresholds.

When stratified by lesion depth, performance patterns were broadly similar for enamel and dentin lesions. The enamel–dentin junction was selected as the analytical boundary because it represents a biologically and radiographically meaningful threshold commonly used in ICDAS-based validation studies. In both categories, specificity and NPV remained high, whereas sensitivity was moderate and PPV very low. These findings suggest that the AI software is more reliable in excluding disease than in confidently confirming lesion presence, regardless of lesion depth.

This consideration is more critical in low-prevalence populations, such as the one in the present study, where PPV can be low and false-positive results can give rise to overtreatment. Therefore, high specificity is required to ensure accurate identification of sound surfaces [33].

Our aggregate performance (sensitivity ~ 73%, specificity ~ 94%, κ ~ 0.638) aligns with independent Diagnocat™ validation on intraoral radiographs, where per-surface sensitivity ~ 0.51–0.76 and specificity ~ 0.88–0.97 were reported [21]. In the broader AI-for-caries literature on bitewings, meta-analyses report high pooled sensitivity and specificity (e.g., 0.94/0.91 and 0.87/0.89) with model- and dataset-dependent variability [15, 16]. Our results fall within the lower-middle range for sensitivity but upper range for specificity, which likely reflects a stringent reference standard (calibrated experts using ICDAS constructs [8, 9]) and real-world images rather than curated datasets. The high NPV we observed is consistent with meta-analytic conclusions that AI is generally reliable at excluding disease on bitewings; however, variability in PPV across studies highlights the need for cautious interpretation of positive findings [15, 16]. Another recent review and meta-analysis by Luke et al. similarly concluded that many AI models demonstrate high sensitivity and specificity in caries detection, sometimes exceeding 90% on both metrics, but with substantial variability across dasasets and lesion definitions [34]. Diagnocat™ results are mid-to-high for specificity and mid-range for sensitivity under these constraints.

Comparative AI-vs-human studies reinforce these patterns. In a randomized controlled trial on intraoral radiographs, an AI slightly exceeded dentists in both sensitivity and specificity (~ 88% and 91% vs. 84% and 88%), with accuracy 89% vs. 86% [35]. Conversely, AssistDent on bitewings increased enamel-only sensitivity for dentists but reduced specificity, illustrating a sensitivity–specificity trade-off when AI prompts are used for early lesions [24]. Our findings demonstrate that Diagnocat™ exhibited high specificity and negative predictive value, but relatively lower sensitivity and positive predictive value. This indicates that the AI software is more reliable at correctly identifying sound surfaces than detecting all caries lesions, particularly subtle demineralizations. Consequently, while the software can provide useful guidance, it may miss early lesions and does not replace clinical judgement.

Beyond adult intraoral radiography, other recent work further contextualizes our findings. A pediatric pilot study using CNNs for approximal caries on periapical radiographs showed promising discrimination in children aged 5–12 years, supporting AI’s utility across age groups [36]. In 3D imaging, AI-assisted CBCT caries detection has also been explored. A decision-support system evaluated on 500 CBCT volumes enhanced reader confidence and diagnostic performance [31]. More broadly, the Diagnocat™ platform has been iteratively developed and clinically studied in CBCT workflows since its early deployments; a 2021 investigation described a multi-module deep learning system (including caries and periapical modules) that improved dentists’ diagnostic performance in clinical settings [37]. A recent technical report evaluated Diagnocat^tm^’s radiological report function on CBCT and suggested satisfactory interpretive performance, while emphasizing the need for further validation [38]. These studies, although on CBCT rather than bitewings, show an ongoing refinement trajectory for Diagnocat™ across modalities; however, its algorithmic architecture and training datasets are proprietary and not publicly disclosed, limiting external reproducibility. This lack of transparency regarding Diagnocat™’s algorithmic structure and training datasets reflects a broader challenge in commercial AI systems, where insufficient technical disclosure reduces explainability and limits scientific validation.

Strengths of our study include the use of real-world bitewings, ICDAS-informed calibration [8, 9], surface level analysis, and blinded human vs. AI readings—factors that likely improved internal validity. Limitations include a relatively modest sample size, which may constrain generalizability; however, at the surface level, the 1540 examined surfaces were considered sufficient. Furthermore, the random selection of radiographs from a dental clinic’s patient pool enhances the representativeness of the sample for the general population. Moreover, variations in radiographic equipment, imaging protocols, and patient positioning across clinical environments may affect reproducibility, meaning that results obtained in our dataset cannot be assumed to generalize without caution. All bitewing radiographs were exported in JPEG format and reviewed on 14-inch, 1360 × 768 resolution, 8-bit grayscale monitors under ambient light conditions. While this setup may reduce the visibility of subtle radiolucencies compared to higher-resolution, diagnostic-grade monitors, it reflects the imaging system available at our university and simulates real clinical conditions, as these are the same computers used by students in dental operatories. Also, identical image formats and viewing conditions were applied for both human observers and the AI software, minimizing the risk of systematic bias. Future research with larger, multi-center datasets, ideally encompassing diverse populations and imaging systems, will be essential to confirm the robustness and generalizability of our findings. Another limitation is the absence of a true biologic gold standard such as histological validation of caries. We defined ground truth by expert radiographic consensus, which is a practical and common approach in imaging studies but not infallible. Thus, the AI’s sensitivity and specificity are contingent on radiographic detection only, not actual lesion activity or cavitation. Along these lines, we did not incorporate clinical data (such as visual inspection findings, patient caries risk assessment, etc.) into the reference standard. In practice, dentists combine radiographic findings with clinical context; our study isolated the radiographic task to evaluate the AI, but this means some “lesions” identified (by both AI and experts) might have been misdiagnosed in comparison to a real clinical decision-making scenario. Another limitation is that we evaluated only one AI software (Diagnocat™) and a specific version of its algorithm. AI software can improve over time with new training data and algorithm updates; our results are a snapshot of the technology’s capability at the time of the study. It’s possible that newer versions of Diagnocat™ or other AI platforms have achieved higher sensitivity without sacrificing specificity, or vice versa. We did not compare Diagnocat™ to other available caries detection AIs in this study, so we cannot extrapolate these findings to all systems on the market. Finally, our study focused purely on diagnostic performance; we did not assess workflow integration or real-time use of the AI in clinic. It remains to be seen how dentists interact with such software in practice – for example, whether too many false positive alerts might cause alert fatigue, or how much time an AI actually saves during interpretation.

Future research should include prospective clinical trials evaluating AI-assisted caries detection in routine practice, including its impact on diagnostic accuracy, treatment decisions, and potential overtreatment. In cases of equivocal findings on 2D radiographs, adjunctive 3D imaging (CBCT) has been suggested as a possible validation approach [20]; however, its use must remain strictly justified and in accordance with ALARA/ALADA principles [39], and should not be employed routinely for caries diagnosis [40–42]. Such investigations will help refine the responsible integration of AI tools alongside clinician judgement.

## Conclusions

Diagnocat™ demonstrated high specificity and substantial agreement with expert consensus on bitewing radiographs, with moderate sensitivity overall and relatively worse performance for dentin than enamel lesions; however, these differences were not statistically significant. The system’s high NPV indicates reliable exclusion of disease on AI-negative surfaces, whereas low PPV, significant cluster-robust McNemar type differences, and low sensitivity underscore the need for clinician confirmation of AI-positive findings. Based on both our results and the broader evidence, we advocate a conservative, adjunctive role for AI in caries detection: it can assist clinicians by providing a second opinion and improving the consistency of radiographic interpretation, but it cannot replace the need for expert clinical judgment. Used wisely, AI has the potential to streamline diagnostics, standardize caries assessments, and possibly improve early detection, which could benefit patient outcomes. Yet, the dentist should remain in control, verifying AI-identified lesions and integrating clinical context before making treatment decisions, thereby harnessing the strengths of AI while safeguarding against its limitations. Continued improvements in AI algorithms, larger validation studies (such as incorporating different populations and radiographic techniques), and real-world trials will further clarify how these tools can be optimally integrated into dental practice.

## Electronic Supplementary Material

Below is the link to the electronic supplementary material.

Supplementary Material 1

## Data Availability

Data can be provided upon request.
