# Supplementary material for: Evaluating AI diagnostic accuracy in approximal dental caries detection on bitewing radiographs
Source: Clin Oral Investig. 2026 Apr 29;30(5):207. doi: 10.1007/s00784-026-06882-z (PMC13124809; doi:10.1007/s00784-026-06882-z)
Supplement: Supplementary file 1 — Supplementary Material 1 [file 784_2026_6882_MOESM1_ESM.docx]

**Supplemental Table 1.** Diagnostic accuracy measures and corresponding 95% confidence intervals (95% CI) for the identification of caries lesions by the AI software, compared with the reference standard dataset.

| Comparisons | Sensitivity (95%CI) | Specificity  (95%CI) | Positive predictive value (95%CI) | Negative predictive value (95%CI) |
| --- | --- | --- | --- | --- |
| Identification of caries lesions by AI software compared to the reference standard dataset | 73.1%  (95% CI: 65.9%–79.9%) | 94.3%  (95% CI 92.4%–96.0%) | 64.7%  (95% CI 57.7%–71.5%) | 96.1%  (95% CI 94.7%–97.3%) |
| Identification of enamel caries lesions by AI software compared to the reference standard dataset | 73.3% (95% CI 62.8%–82.7%) | 92.9%  (95% CI 91.0%–94.7%) | 42.0%  (95% CI 33.1%–51.2%) | 98.0%  (95% CI 97.1%–98.8%), |
| Identification of dentin caries lesions by AI software compared to the reference standard dataset | 72.8% (95% CI 61.8%–83.8%) | 92.8% (95% CI 90.9%–94.6%) | 39.2% (95% CI 31.6%–46.6%) | 98.2% (95% CI 97.2%–99.0%) |
